# Synthesis, characterization and photoinduced curing of polysulfones with (meth)acrylate functionalities

**DOI:** 10.3762/bjoc.6.56

**Published:** 2010-06-01

**Authors:** Cemil Dizman, Sahin Ates, Lokman Torun, Yusuf Yagci

**Affiliations:** 1 Chemistry Institute, TUBITAK Marmara Research Center, Gebze, Kocaeli 41470, Turkey; 2 Department of Chemistry, Istanbul Technical University, Maslak, Istanbul 34469, Turkey

**Keywords:** acrylates, photoinitiated polymerization, polysulfone, UV-curable oligomers

## Abstract

The UV-curable telechelic polysulfones with (meth)acrylate functionalities were synthesized by condensation polymerization and subsequent esterification. The final polymers and intermediates at various stages were characterized by ^1^H NMR, FT-ATR, and GPC. The oligomeric films prepared from the appropriate solutions containing these telechelics and 2,2-dimethoxy-2-phenylacetophenone (DMPA) as the photoinitiator undergo rapid polymerization upon irradiation forming insoluble networks. The photo-curing behavior was investigated by photo-DSC and the effects of the molecular weight of the polysulfone precursor and type of functionality on the rate of polymerization and conversion were evaluated. Thermal properties of the photochemically cured films were studied by differential scanning calorimeter (DSC) and thermal gravimetric analysis (TGA).

## Introduction

Polysulfones (PSU) show useful properties such as high strength and stiffness even at elevated temperatures, high continuous use and heat deflection temperatures, excellent resistance to hydrolysis by acids and bases, and good dimensional stability even in complex geometric shapes [1]. Despite these exceptional properties, there is need to modify the PSU structure to obtain several desired features. In general polymer modification is the one of main routes to achieve such characteristics [2–3]. There are two ways to functionalize PSUs. The first is postpolymerization modification in which the polymer is modified after polymerization. The second involves direct copolymerization of functionalized monomers [4–6]. An increasing topic of interest concerns cooperation of PSUs with epoxy resins via end group functionalization or blending. Engineering thermoplastics based on PSU have been widely used to overcome the problems associated with the brittleness of epoxy resins [7–11].

Telechelic oligomers are defined as the prepolymers carrying one or more functional end groups. They take part in further polymerization or other reactions through their functional end groups [12]. The functionality of the end groups itself is important. When such groups are bifunctional (e.g. vinyl groups) they can participate in polymerization reactions, yielding graft copolymers or networks; such telechelic polymers are called macromolecular monomers, macromonomers. Their synthesis and modification have been studied in detail and covered by several review articles [13–15].

Photopolymerization is a frequently used process for the conversion of the multifunctional monomers into insoluble networks which are effective in various industrial fields such as, films, inks, coatings; photoresists, etc. [14]. The process is based upon the irradiation of the organic materials with light to initiate the reaction [15]. Compared to thermal polymerization, the corresponding photochemical processes have several advantages including increasing manufacturing capacity, low energy requirements, decreasing working area, production-line adjustability, low temperature processing, non-polluting and solvent-free formulations, and uncomplicated designed system. Organic materials (monomers, oligomers, polymers) with photoinitiators can be used in UV-curing systems. There are several photoinitiators acting in the UV and visible range capable of inducing rapid polymerization to form insoluble networks [16]. Because of their high reactivity leading to fast polymerization [17], multifunctional (meth)acrylates are the most commonly used monomers for many applications [18–22]. The activity of the (meth)acrylates depends on their structural properties such as the type and flexibility of incorporated molecule, number of functional groups, the presence of heteroatoms, chain length, and hydrogen bonding etc. [23–26].

In the present work, we report the preparation and characterization of UV curable (meth)acrylate telechelics with polysulfone backbones. The curing behavior of these telechelics was studied by photo-DSC with 2,2-dimethoxy-2-phenylacetophenone (DMPA) as the photoinitiator. As shown below, the rigid aromatic polysulfones with different molecular weights were deliberately used so as to demonstrate the structural and molecular weight effects on the curing behavior. Finally, the durability of the cross-linking material was investigated by TGA.

## Results and Discussion

**Scheme 1:**
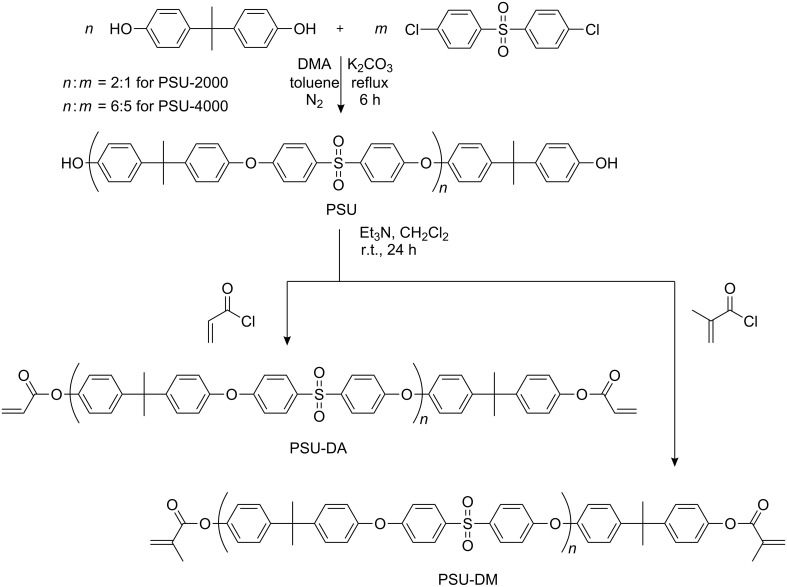
Synthesis of polysulfone macromonomers.

UV-Curable PSU telechelics were synthesized by condensation polymerization and subsequent esterification. First of all, PSU-2000 and PSU-4000 were synthesized by condensation polymerization according to the procedure described in the literature [27]. Monomer concentrations were adjusted to yield oligomers possessing phenolic groups at both ends. Polysulfone macromonomers were then synthesized by esterification of the oligomers obtained with acryloyl chloride and methacryloyl chloride in the presence of Et_3_N as the base. The overall procedure is presented in Scheme 1.

**Figure 1:**
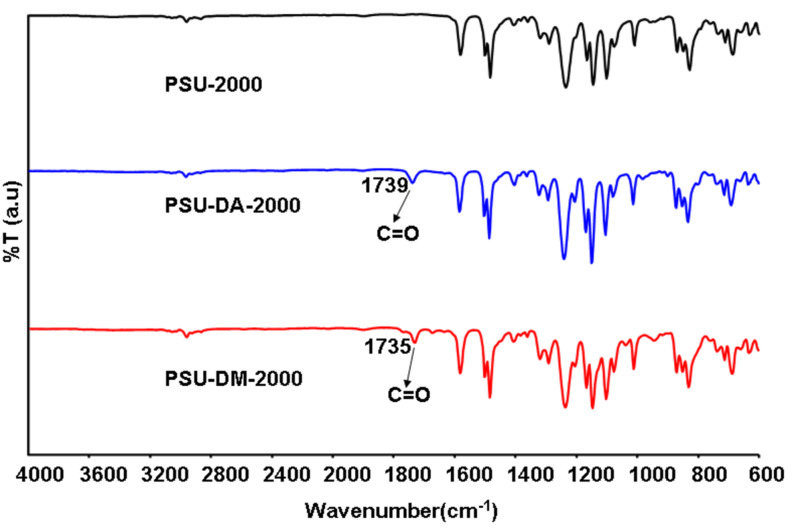
FT-IR spectra of PSU-2000, PSU-DA-2000 and PSU-DM-2000.

The characterization of the synthesized oligomers was carried out by using FTIR-ATR, ^1^H NMR, GPC, DSC, photo-DSC and TGA. FTIR-ATR data shows the characteristic bands for the polyether sulfone backbone. The new broad but weak peak around 3435 cm^−1^ indicates the presence of phenolic end groups. Attachment of polymerizable acrylate and methylacrylate functional groups through the esterification process was evidenced by the disappearance of this peak and the formation of the new ester carbonyl peak at around 1735 cm^−1^ (Figure 1).

**Figure 2:**
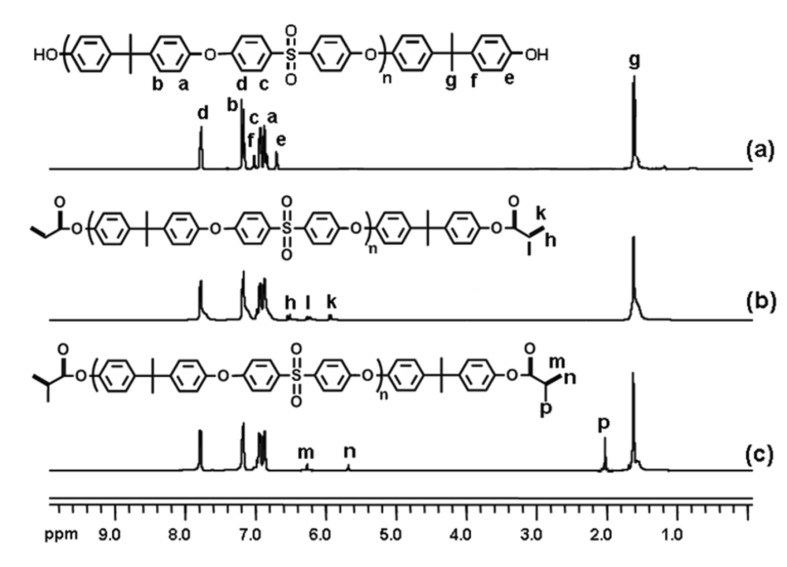
^1^H NMR spectra of PSU-2000 (a), PSU-DA-2000 (b) and PSU-DM-2000 (c) in CDCl_3_.

The structures of the macromonomers were further confirmed by ^1^H NMR analysis. As can be seen from Figure 2, where the ^1^H NMR spectra of the precursor polymer and telechelics are presented, the methyl group belonging to bisphenol A appears in all the spectra at 1.69 ppm. The shifts between 6.70 and 7.86 ppm correspond to the aromatic protons of the poly(ether sulfone) backbone. The phenolic protons were not observed, probably due to the relatively high molecular weight of the precursor polymers. Distinctively, the aromatic protons of the terminal benzene ring appeared at 6.77 and 7.07 ppm as relatively weak signals. Successful macromonomer formation was confirmed by the appearance of the new peaks at around 6.15 (d), 6.50 (t) and 6.75 (d) ppm for the acrylate and 6.15 (s) and 6.45 (s) ppm for methacrylate groups, respectively. Notably, in both cases, the aromatic protons were down field shifted. End chain aromatic protons overlap with the other aromatic protons. The signal at 2.02 (s) ppm was assigned to the methyl group of the methacrylate functionality.

The molecular weight characteristics of the polymers with respect to the synthesis conditions are presented in Table 1. As the functionalized PSUs were intended to be used in the ultimate photocuring step, the conditions of polycondensations were chosen so as to obtain relatively low molecular weight polymers with phenolic end group functionality combined with a satisfactory conversion. The efficiency of functionalization was confirmed by ^1^H NMR spectrum by using the integration ratio of the protons corresponding to the (meth)acrylic groups to that of the methyl protons of the repeating unit. Almost quantitative functionalization was attained in both cases. Notably, the molecular weights calculated by ^1^H NMR in general agree well with the measured values. Moreover, general agreement between the molecular weight of the final telechelic polymers and that of the precursor PSU obtained by GPC also confirms efficient esterification. The observed increase in the molecular weight is due to the additional acrylate and methacrylate moieties incorporated.

**Table 1:** Synthesis^a^ and molecular weight characteristics of polysulfones.

Polymer	Bisphenol A / Chlorosulfone (mol/mol)	Yield^b^	*M*_n_^c^ _(GPC)_ (g/mol)	PDI	*M*_n_^d^ _(NMR)_ (g/mol)	Acrylates		Methacrylates	
						*M*_n_^c^ _(GPC)_ (g/mol)	*M*_n_^d^ _(NMR)_ (g/mol)	*M*_n_^c^ _(GPC)_ (g/mol)	*M*_n_^d^ _(NMR)_ (g/mol)

PSU-2000	2/1	65%	1850	1.45	2150	2198	2396	2120	2323
PSU-4000	6/5	71%	4400	1.51	4000	4605	4228	–	–

^a^Reaction temperature: 170 °C, time: 6 h. ^b^Determined gravimetrically. ^c^Number average molecular weight determined from GPC measurements based on polystyrene standards. ^d^Calculated by using ^1^H NMR spectra.

**Figure 3:**
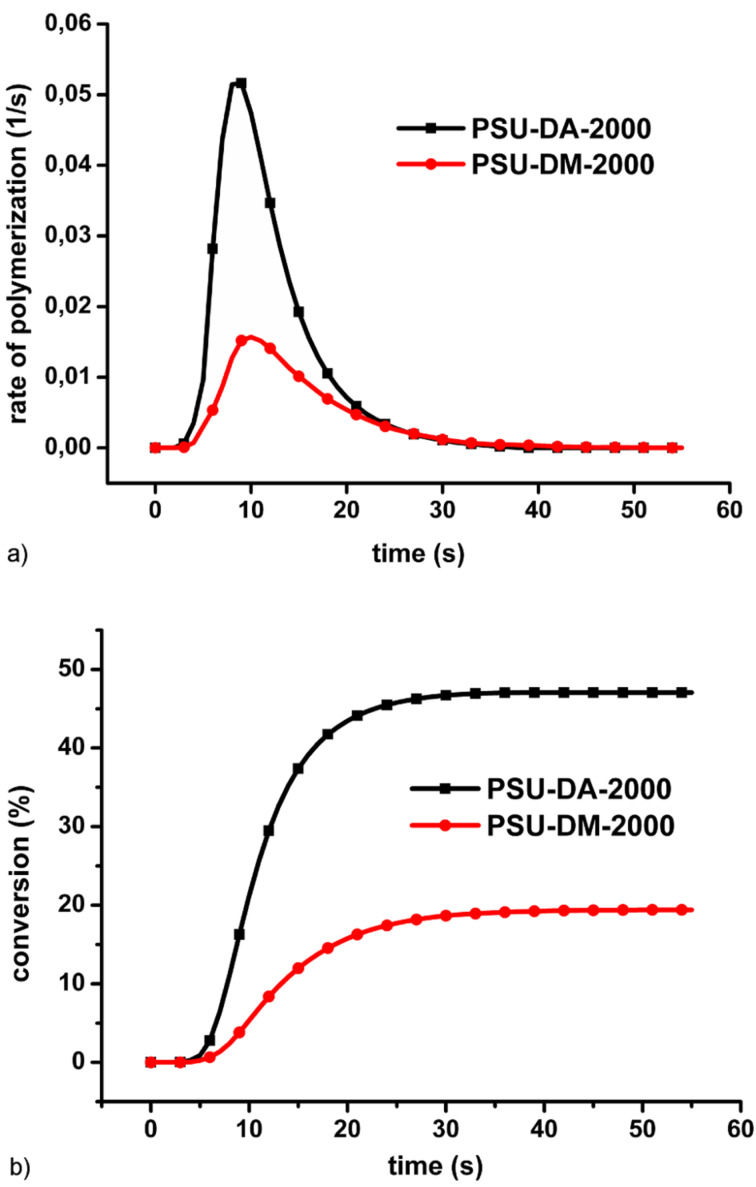
Rate (a) and conversion (b) of photo induced polymerization of PSU-DA-2000 and PSU-DM-2000.

**Figure 4:**
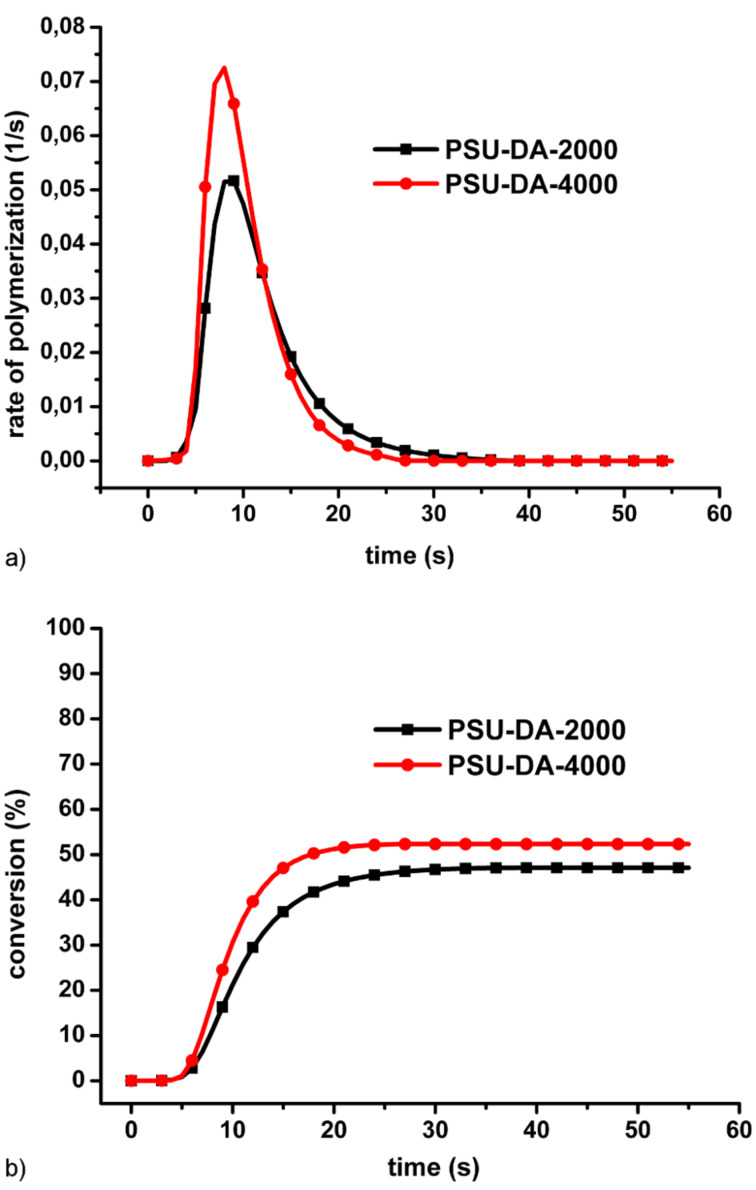
Rate (a) and conversion (b) of photo induced polymerization of PSU-DAs with different molecular weights.

Kinetic studies concerning photopolymerization of the macromonomers were performed by photo-DSC. The results are shown in Figure 3 and Figure 4. The rate of photopolymerization vs. time plots for both PSU-DA-2000 and PSU-DM-4000 exhibit no plateau region indicating the absence of the rapid auto acceleration at the very beginning of the reaction. This behavior may be due to factors related to the cross-linking nature of the samples and solid-state measurements. Since the obtained telechelics exhibit high melting points and are solid at room temperature, free standing films can easily be prepared. The photopolymerization under these conditions leads to a suppressed center of coil diffusion resulting in rapid auto acceleration [28]. The results also indicate that conversions are lower than 50%. Although the oligomers possess long flexible chains, due to their vitreous nature dense cross-linked network formation occurs which decreases the amount of reacted double bonds significantly [29]. Figure 3 also shows that the conversion and polymerization rate of the acrylate derivative is considerably higher than that of the methacrylate macromonomer. This difference may be due to the α-methyl group present in the monomer which stabilizes the propagating radical. These results are consistent with the literature data for the thermal initiated polymerization that gives a difference by a factor of approximately 5 in the polymerization rates at room temperature [30].

The cross-linking capability of the oligomers increased with the molecular weight of the macromonomer as a result of the increased flexibility of the longer chains. Therefore, PSU-DA-4000 displays a slightly higher conversion and a faster rate of polymerization (Figure 4) [31].

**Figure 5:**
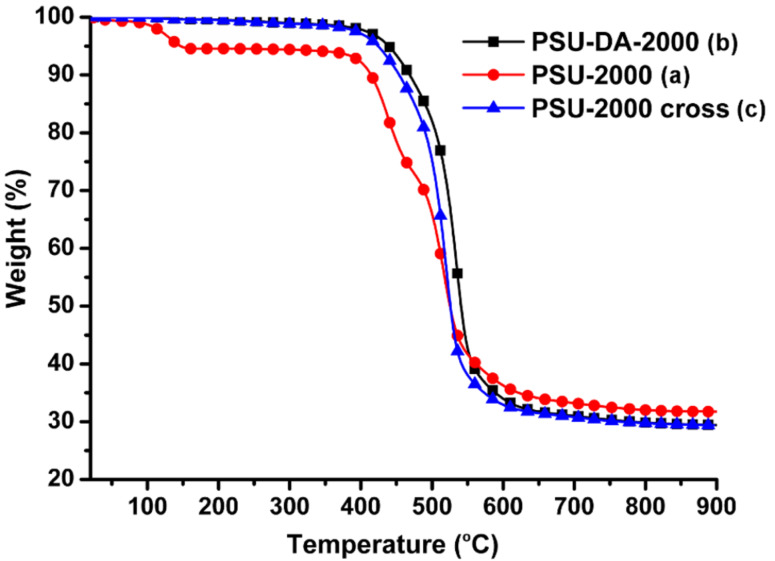
TGA thermograms of the precursor oligomers (PSU-2000) (a), and macromonomer, PSU-DA-2000 before (b), and after curing (PSU-DA-2000 X) (c).

TGA thermograms of photochemically cured and precursor oligomers are shown in Figure 5. As can be seen, the initial oligomers show a small weight loss up to 200 °C. This degradation can be attributed to the elimination of water. Similar weight losses have been observed for other hydroxyl-containing polymers [32]. Major degradation of the oligomers began at around 400 °C. The overall thermal stability of the polymers is similar.

**Figure 6:**
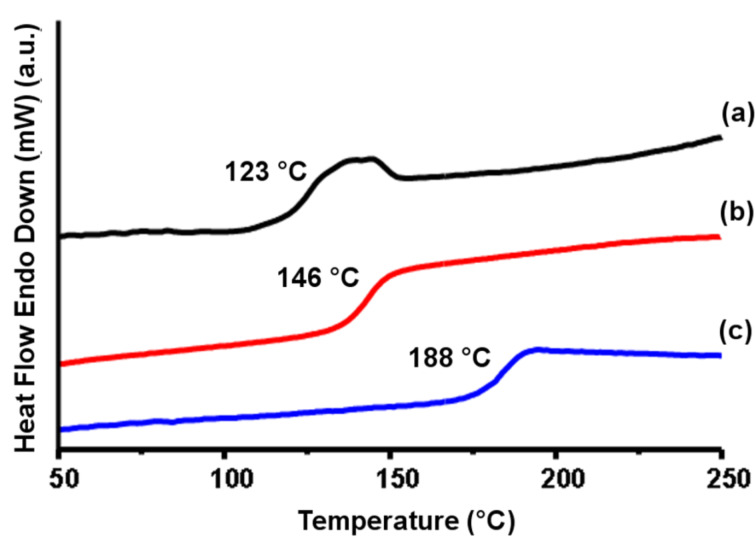
DSC results of the precursor oligomer (PSU-2000) (a), and macromonomer, PSU-DA-2000 before (b), and after curing (PSU-DA-2000 X) (c).

As can be seen from Figure 6, the glass transition temperature (*T*_g_) of the acrylate macromonomer is more than 20 °C higher than that of its precursor polymer because of the structural compatibility between the end groups and the inner backbone. Interestingly, the corresponding UV-cured macromonomer exhibits a much higher *T*_g_ (188 °C) which is almost the same *T*_g_ as commercially available high molecular weight PSUs such as UDEL-PSU (*M*_n_ = 30000). This behavior indicates that the properties of the high molecular weight PSUs can be attained even with oligomeric macromonomers as a result of extended chain length by UV induced cross-linking.

In conclusion, we have synthesized two types of PSU macromonomers by step-growth polymerization and subsequent esterification processes, and investigated their photocuring behavior. The effects of the molecular weight of the PSU precursor and type of functionality on the rate of polymerization and conversion were evaluated. The thermal stability of the photochemically cross-linked polymers indicates that these oligomers may be important components of UV curable formulations for obtaining networks that could have application in coatings and membranes.

## Experimental

### Materials

Bisphenol A and bis(*p*-chlorophenyl) sulfone (Hallochem Pharma Co. Ltd, China), methanol (Merck), dimethylacetamide (DMA, 99%, Merck) were used without any purification. Dichloromethane (99%, Aldrich), chloroform (+99%, Aldrich), acryloyl chloride (+97%, Merck), methacryloyl chloride (+97%, Merck) were used as received. 2,2-Dimethoxy-2-phenylacetophenone (DMPA, 99%, Across) was also used without any additional treatment.

### Characterization

^1^H NMR spectra of 5–10% (w/w) solutions of the intermediates and final polymers in CDCl_3_ with Si(CH_3_)_4_ as an internal standard were recorded at room temperature at 250 MHz on a Bruker DPX 250 spectrometer. Fourier transform infrared-Attenuated Total Reflectance (FTIR-ATR) spectra were recorded on a Perkin-Elmer FT-IR Spectrum One B spectrometer with a Universal ATR accessory equipped with a single reflection diamond crystal. Solid oligomers were placed over the ATR crystal and maximum pressure was applied using the slip-clutch mechanism. Differential scanning calorimetry (DSC) was performed on a Perkin-Elmer Diamond DSC. Molecular weights and polydispersities of the linear oligomers were measured by gel permeation chromatography (GPC) employing an Agilent 1100 instrument equipped with a differential refractometer with tetrahydrofuran as the eluent at a flow rate of 0.3 mL min^−1^ at 30 °C. Molecular weights were determined using polystyrene standards. Thermal gravimetric analysis (TGA) was performed on Perkin–Elmer Diamond TA/TGA with a heating rate of 10 °C min under nitrogen flow.

### Preparation of the oligomers

#### General procedure for the synthesis of polysulfone oligomer

Bisphenol A (40 g, 175 mmol), bis(*p*-chlorophenyl) sulfone (25.16 g, 87.6 mmol) and dried potassium carbonate (25.39 g, 183.6 mmol) were added to 400 mL DMA (dimethyl acetamide) and 50 mL toluene in a 2000 mL, 2 necked round bottom flask, fitted with a condenser, nitrogen inlet, a Dean and Stark trap and an overhead mechanical stirrer. The reaction mixture was heated under reflux at 150 °C for 4 h with water removal. The reaction was stopped after about 2 h and cooled to room temperature. The solution was filtered to remove most of the salts and poured into a mixture of methanol andwater (4:1). The precipitated polymer was filtered, and washed several times with water in order to remove the remaining salts and impurities. Finally, the polymer was washed with methanol and dried in a vacuum oven at 60 °C for about 12 h to give PSU-2000 oligomer (42.2 g).

IR (ATR, cm^−1^): 3435 (-OH), 3200–3000 (Ar), 2975 (-CH_3_ sym-), 2945 (-CH_3_ asym-), 1322 and 1293 (O=S=O asym-), 1240 (C-O-C), 1175 and 1151 (O=S=O sym-) and 1014 (Ar).

^1^H NMR (CDCl_3_, ppm): δ = 7.85 (16H), 7.26 (16H), 7.07 (4H), 7.00 (16H), 6.94 (16H), 6.75 (4H), 1.69 CMe_2_ (30H).

A similar procedure using appropriate ratios of the monomers was used for the synthesis of PSU-4000.

#### Synthesis of polysulfone diacrylate (PSU-DA)

PSU-2000 (5 g, 2.86 mmol) was added to 20 mL CH_2_Cl_2_ in a 50 mL, two necked round bottomed flask fitted with a condenser and argon inlet. The flask was placed in an ice bath and the contents stirred for about 5 min. Excess triethylamine (Et_3_N) 2.0 mL was added followed by excess acryloyl chloride (1.15 mL, 14.3 mmol) dissolved in 5 mL CH_2_Cl_2_ which was added slowly to the reaction flask over a 10 min period. The reaction mixture was stirred for 24 h then filtered to remove the salts formed and poured into methanol to precipitate the acrylate oligomer. The precipitated oligomer was filtered and washed several times with water to remove the remaining salts and impurities. Finally, the polymer was washed with methanol and dried in a vacuum oven at room temperature for about 12 h to give PSU-DA-2000 macromonomer (5 g).

IR (ATR, cm^−1^): 3200–3000 (Ar), 2968 (-CH_3_ sym-), 2875 (-CH_3_ asym-), 1739 (-C=O), 1322 and 1293 (O=S=O asym-), 1238 (C-O-C), 1175 and 1151 (O=S=O sym-) and 1014 (Ar).

^1^H NMR (CDCl_3_, ppm): δ = 7.76 (16H), 7.16 (16H), 7.02 (4H), 6.94 (16H), 6.86 (16H), 6.84 (4H), 6.53–6.51 (2H) and 6.24–6.22 (2H) (CH2=), 5.94–5.92 (2H) (=CH-), 1.62 CMe_2_ (30H).

The same procedure was applied for the synthesis of PSU-DA-4000.

#### Synthesis of polysulfone dimethacrylate (PSU-DM)

For the preparation of PSU-DM-2000, a similar procedure as described for the synthesis of acrylate functional macromonomers was followed with methacryloyl chloride.

IR (ATR, cm^−1^): 3200–3000 (Ar), 2969 (-CH_3_ sym-), 2875 (-CH_3_ asym-), 1735 (-C=O), 1323 and 1295 (O=S=O asym-), 1243 (C-O-C), 1175 and 1151 (O=S=O sym-) and 1014 (Ar).

^1^H NMR (CDCl_3_, ppm): δ = 7.78 (16H), 7.17 (16H), 6.94 (16H), 6.87 (16H), 6.26 (2H) and 5.76 (2H), 1.98 (C=-Me) (6H), 1.63 CMe_2_ (30H).

### Preparation of Photo-curable formulations

Formulations containing macromonomers (0.003 g) and DMPA (2 mol %) in 250 µL chloroform were prepared from appropriate stock solutions. The mixture was then dropped onto an aluminum pan and the solvent allowed to evaporate completely. The film samples were placed into the sample cell of photo-DSC instrument.

### Photocalorimetry (Photo-DSC)

The photo-differential scanning calorimetry (Photo-DSC) measurements were carried out by means of a modified Perkin-Elmer Diamond DSC equipped with a high pressure mercury arc lamp (320–500 nm). A uniform UV light intensity was delivered across the DSC cell to the sample and reference pans. The intensity of the light was measured as 53 mW cm^−2^ by a UV radiometer capable of broad UV range coverage. The mass of the sample was 3 mg, and the measurements were carried out in an isothermal mode at 30 °C under a nitrogen flow of 20 mL min^−1^. The heat liberated in the polymerization was directly proportional to the number of acrylate groups reacted in the system. By integrating the area under the exothermic peak, the conversion of the acrylate groups (*C*) or the extent of the reaction was determined according to Equation 1:

[1]
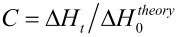


where Δ*H_t_* is the reaction heat evolved at time *t* and Δ*H*_0_^theory^ is the theoretical heat for complete conversion. Δ*H*_0_^theory^ = 86 kJ mol^−1^ for an acrylic double bond [33]. The rate of polymerization (*R*_p_) is directly related to the heat flow (d*H*/d*t*) by Equation 2:

[2]
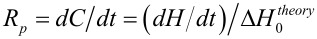

